# Supplementary material for: The relationship between body condition, body composition, and growth in amphibians
Source: PLoS One. 2025 Apr 23;20(4):e0320954. doi: 10.1371/journal.pone.0320954 (PMC12061418; doi:10.1371/journal.pone.0320954)
Supplement: S1 File — S1 Appendix. Details of quantitative magnetic resonance protocol for determining body composition in 3 species of amphibians. S1 Fig. Locations of capture sites in Montana, USA for studying body composition of three species of amphibians (all sites; square, circle, and triangle), as well as growth of Columbia spotted frogs (Jones Pond only; square). Made using Natural Earth. S2 Fig. Percent body fat (a) and scaled body fat (b) as measured by quantitative magnetic resonance, by snout-vent length in Columbia spotted frogs captured in Montana, USA. The relationship (Pearson’s correlation r) of percent fat and scaled fat to snout-vent length varied markedly between adults (males [M] and females [F]) and juveniles (unknown sex [U]), so juveniles were removed from analyses of body composition and growth. Shaded areas are 95% confidence intervals for the linear relationship. S3 Fig. Distributions of growth in mm/day, snout-vent length (SVL), and four body condition indices (body mass index, scaled mass index, residual index, and Fulton’s index) as measured in Columbia spotted frogs in summers of 2020–2023 (n = 743 SVL and body condition measurements, n = 234 growth measurements). S4 Fig. Percent body fat by residual index of female (a) and male (b) Columbia spotted frogs at Jones Pond, Montana. Measurements of body fat were taken via quantitative magnetic resonance across three months in summers 2021 and 2022. Shaded areas are 95% confidence intervals for the linear relationship. (DOCX) [file pone.0320954.s001.docx]

The relationship between body condition, body composition, and growth in amphibians. PLOS One

Hinderer, R. K., B. R. Hossack, and L. A. Eby.

**Supporting Information**

S1 Appendix: Details of quantitative magnetic resonance protocol for determining body composition in 3 species of amphibians.

We first gently dried amphibians with a clean paper towel, then measured snout-vent length (SVL) to the nearest 1mm with a ruler and mass to the nearest 0.01g with a bench scale. We set the EchoMRI-500 (www.echomri.com, Houston, Texas, USA) quantitative magnetic resonance (QMR) analyzer to measure three or five distinct measures of lean mass (including muscle and bone), free water (liquid water found in the bladder or on the surface of an animal), total water (all water, including that within body tissues), and total fat content. The machine rested for 20 seconds between measurements, and did not accumulate (e.g., average) measurements so that we could directly observe variance in body composition measurements. When scans were completed, we returned amphibians to holding containers with dampened substrate and released them to their place of origin within 24 hrs. All holding containers and analyzer specimen tubes were washed or sterilized between animals to reduce the risk of disease transmission. All protocols complied with Montana Fish, Wildlife, and Parks scientific collectors’ permit 2021-066-W and University of Montana Institutional Animal Care and Use Committee protocol 014-21LEECS-031621.

We noted some variability in repeated measurements of animals during QMR scanning. Based on conversations with the manufacturer of the QMR machine, we attributed larger discrepancies in measurements of fat mass to the electronic “noise floor” where readings at very small animal sizes could be affected by environmental electrical interference. To reduce error in body composition measurements, we filtered observations where the measurement of fat mass was > 2 standard deviations from the mean of the three or five measurements taken for the same individual on the same day. This resulted in 77 out of 1,111 total scans being filtered out, including all measurements of 1 Columbia spotted frog, 1 long-toed salamander, and 2 Sierran treefrogs. We averaged the remaining measurements to obtain a single value, per animal, per day for each of the body composition measures.


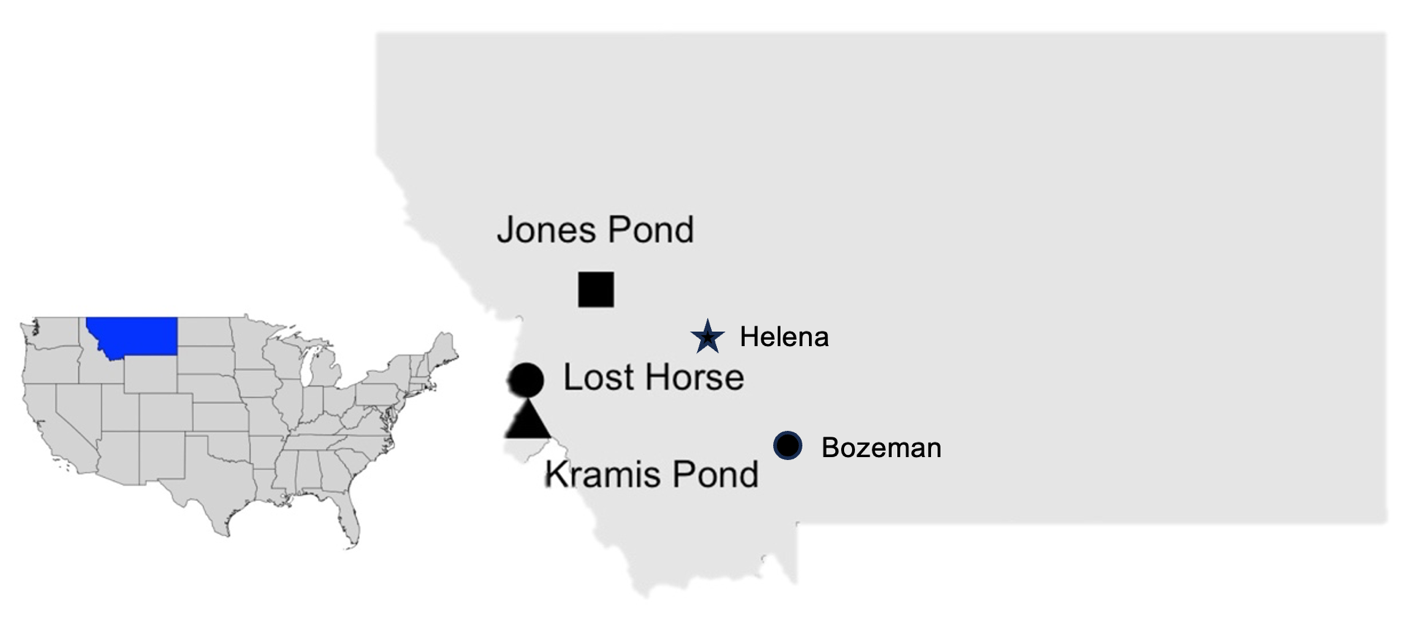


S1 Figure. Locations of capture sites in Montana, USA for studying body composition of three species of amphibians (all sites; square, circle, and triangle), as well as growth of Columbia spotted frogs (Jones Pond only; square). Made using Natural Earth.


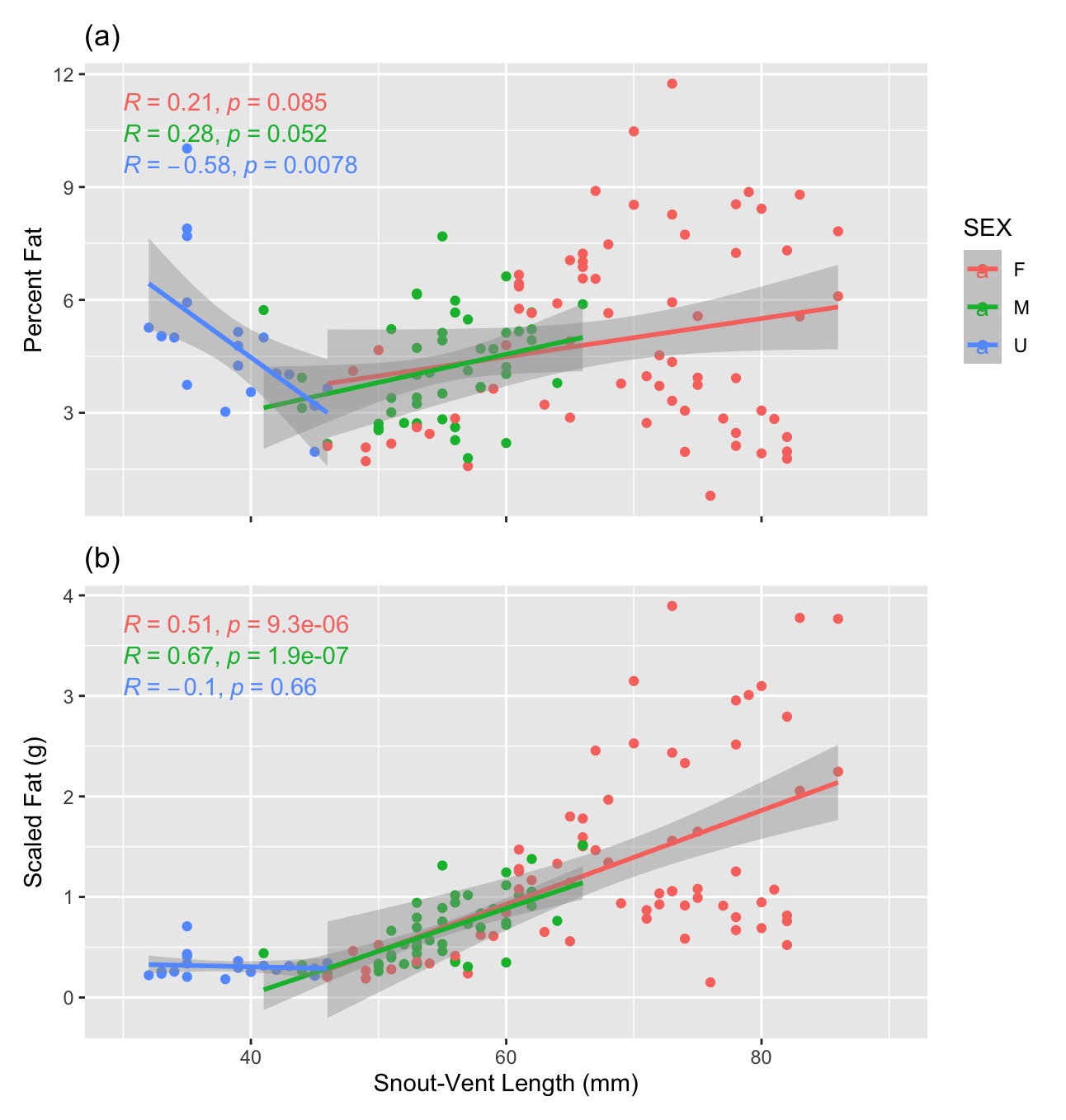


*r =* 0.51, *p* = 0.00

*r* = 0.67, *p* = 0.00

*r* = -0.10 *p* = 0.66

*r =* 0.21, *p* = 0.09

*r* = 0.28, *p* = 0.05

*r* = -0.58, *p* = 0.01

S2 Figure. Percent body fat (a) and scaled body fat (b) as measured by quantitative magnetic resonance, by snout-vent length in Columbia spotted frogs captured in Montana, USA. The relationship (Pearson’s correlation *r*) of percent fat and scaled fat to snout-vent length varied markedly between adults (males [M] and females [F]) and juveniles (unknown sex [U]), so juveniles were removed from analyses of body composition and growth. Shaded areas are 95% confidence intervals for the linear relationship.


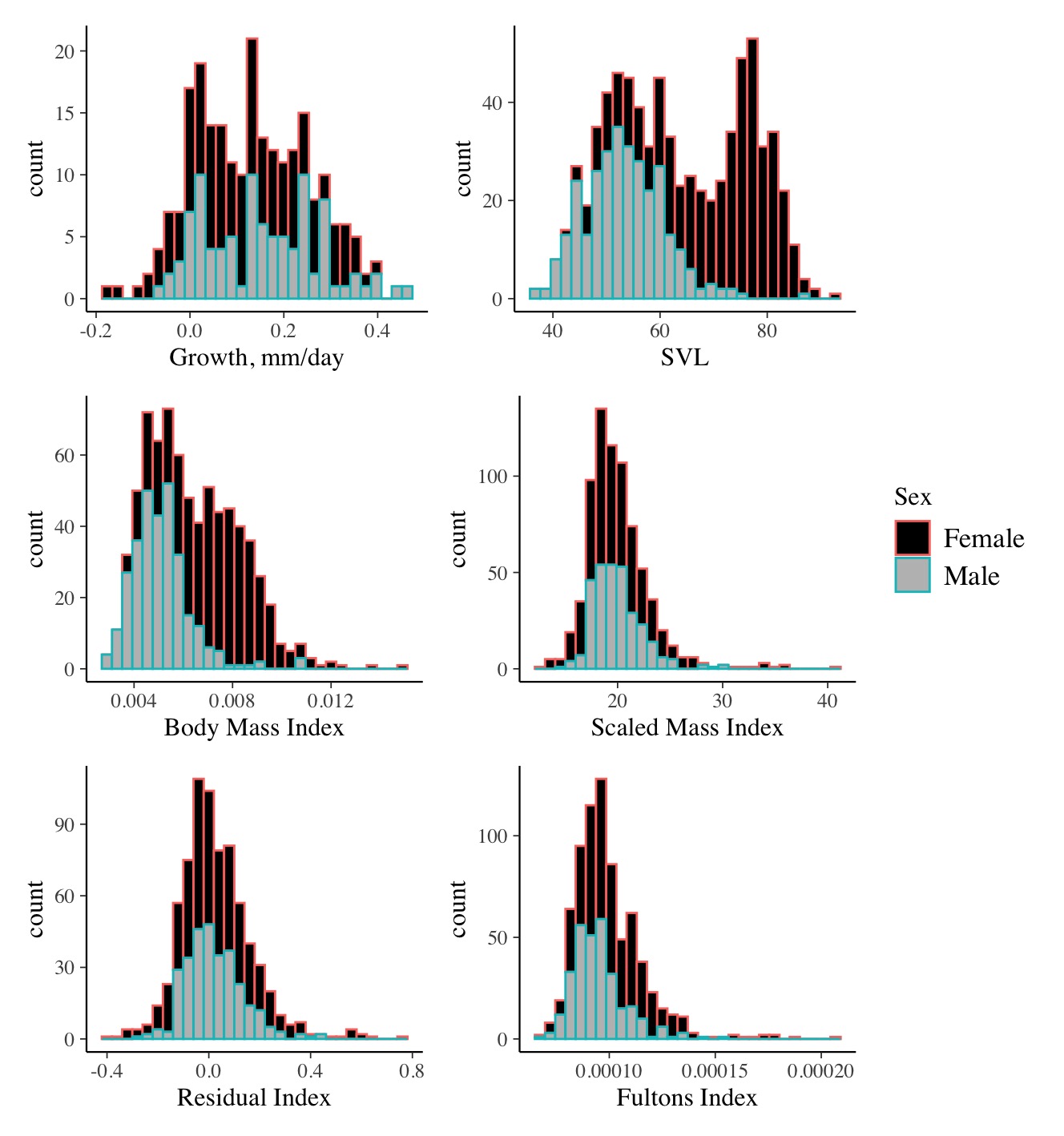


S3 Figure. Distributions of growth in mm/day, snout-vent length (SVL), and four body condition indices (body mass index, scaled mass index, residual index, and Fulton’s index) as measured in Columbia spotted frogs in summers of 2020–2023 (*n* = 743 SVL and body condition measurements, *n* = 234 growth measurements).


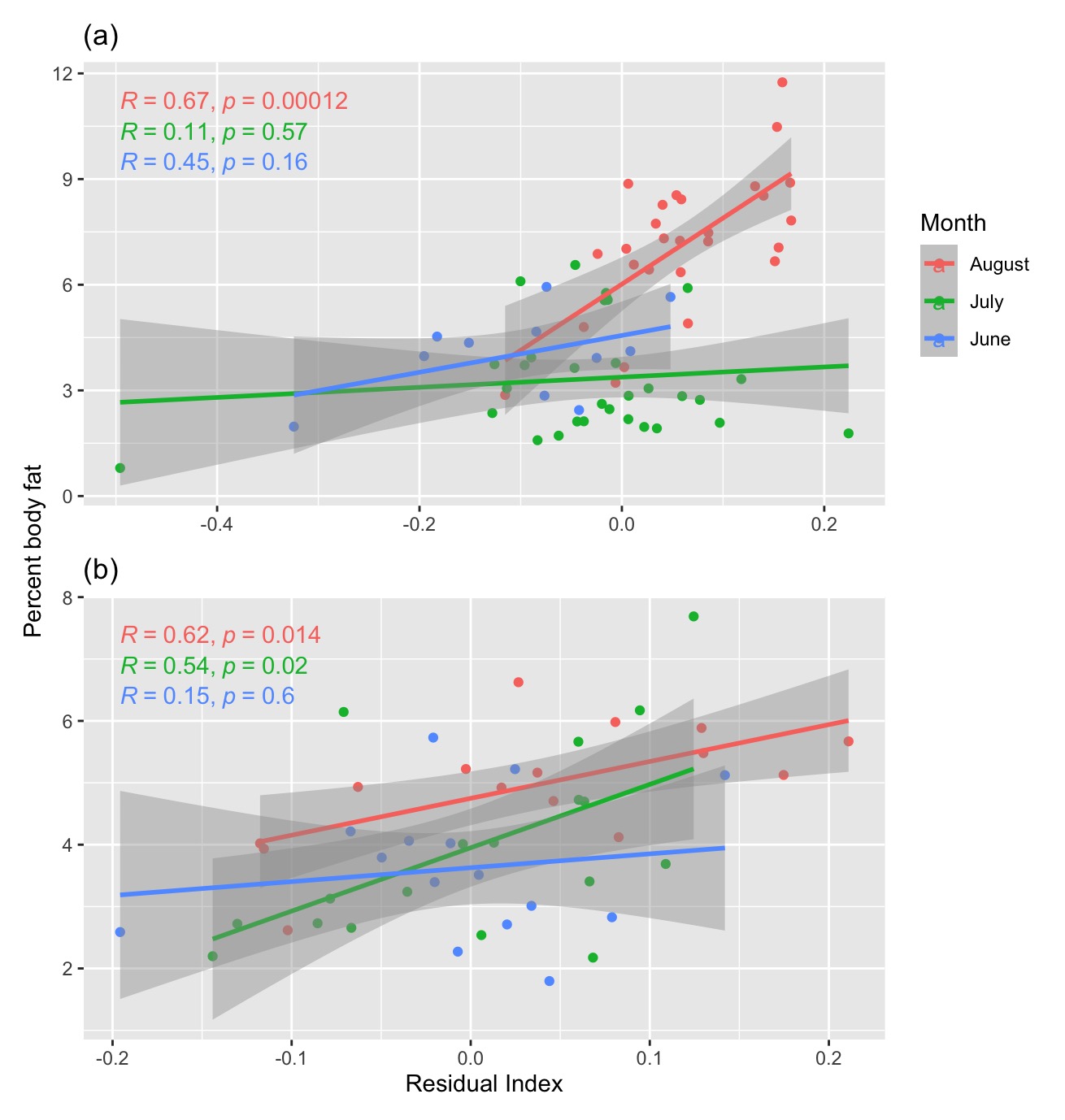


S4 Figure. Percent body fat by residual index of female (a) and male (b) Columbia spotted frogs at Jones Pond, Montana. Measurements of body fat were taken via quantitative magnetic resonance across three months in summers 2021 and 2022. Shaded areas are 95% confidence intervals for the linear relationship.
