# Supplementary material for: The relationship between body condition, body composition, and growth in amphibians
Source: PLoS One. 2025 Apr 23;20(4):e0320954. doi: 10.1371/journal.pone.0320954 (PMC12061418; doi:10.1371/journal.pone.0320954)
Supplement: Attachment — Submitted filename: Response to Reviewers.docx [file pone.0320954.s003.docx]

Our responses to each reviewer point are set off by double-carats (>>) below. Line numbers refer to the “clean” manuscript version without track changes. We made some additional edits based on an internal review, which are also reflected in track changes.

**Reviewer #1:** The manuscript presents a simple but very useful study linking body condition with body composition. While the results and recommendations are useful, I have several concerns regarding the study in general:
1. the sampling period was large covering pre and post reproduction. During reproduction both females and males lose weight. This will bias the results. Please clarify this aspect

>> Sierran treefrogs and long-toed salamanders were captured during their breeding seasons, while Columbia spotted frogs were captured June-August, well after they breed in our area. We had no way to assess whether salamanders or treefrogs had bred prior to capture. However, amphibian surveys often occur during breeding and nonbreeding seasons, and we aimed to make our results useful for practitioners in the field. The fact that some animals may have bred prior to capture should not bias results in any systematic way, as QMR gives a direct measurement of body composition which would reflect any loss of fat, muscle, or water, and loss of mass is reflected in BCI calculations. We have added language to address this point: “Though we had no way to assess whether animals had previously bred shortly prior to capture, this is typical in amphibian population studies which occur before, during, or after the breeding season” (93–95).

2. the sample size for females in treefrogs is very small (4 individuals) to draw any sound conclusions.

>> We agree that the sample size of female treefrogs is quite small. We specifically emphasized this point on lines 261 and 339–340, explaining the lack of relationship observed between allocation of body mass and sex. Elsewhere, both sexes of treefrogs are pooled to avoid making any conclusions from this small sample size.

3. the fact that Fulton's index and BMI are correlated with SVL is not unexpected. This is the main reason the two indeces are seldom used (at least to my knowledge).

>> This is true, and mentioned in the discussion: “Several authors have noted that condition and size are commonly correlated in condition indices based on ratios between length and mass, making it difficult to separate the effects of condition and size” (318–320). We tested the relationship between body composition and these ratio indices, as they are still commonly found in literature from wildlife studies to human health research.

4. considering that a single species of salamander and only two anuran species were used, the conclusion in L292-3 is totally speculative and not supported by the data.

>> We have changed this statement to refer only to the three species in our analysis: “across the three species we studied, despite differences in body morphology” (line 305–307).

My suggestion is to limit the analysis to Columbia spotted frog data. This will provide a solid case study. It could afterwards include other species and allow or a search of patterns and trends.

>> We appreciate this suggestion. However, given the general lack of published research verifying the link between body condition indices and body composition for amphibians, we feel that including all three species is an important contribution to the field.

**Reviewer #2:** This study aims to link 4 values of body condition indices, which are calculated from values easily measurable in the field, to the body composition in 3 species of amphibians, and then test the reflection of these BCIs in the future growth of one of the species. The authors do a good job explaining the importance and goals of their research, describing in detail their methods and results. Following long-term growth rates of individuals in their natural environment is difficult and as such not many studies are available, and the connection with BCI provides us a better choice between various indices.

I have two main comments, which should be easily fixable:
- First, although the study was conducted “during the breeding season through late summer” (line 87), we are given no information related to how data of females full of eggs were treated. Since egg-mass can represent an important part of body mass before egg-laying (even 70-80%), this is likely to introduce an important bias into the values of BCI. An additional related question is: were such animals scanned, and to what type of component would the egg-mass belong? If the answer is simply that all individuals were measured after reproduction ended, this should be clearly stated in the ms.

>> Sierran treefrogs and long-toed salamanders were captured during their breeding seasons, while Columbia spotted frogs were captured during breeding and during June-August, well after they breed in our area. We had no way to assess whether animals had bred prior to capture. However, amphibian surveys often occur during breeding and nonbreeding seasons, and we aimed to make our results useful for practitioners in the field. The fact that some animals may have bred prior to capture should not bias results in any systematic way, as QMR gives a direct measurement of body composition which would reflect any loss of fat, muscle, or water, and loss of mass is reflected in BCI calculations. We have added language to address this point: “Though we had no way to assess whether animals had previously bred shortly prior to capture, this is typical in amphibian population studies which occur before, during, or after the breeding season” (93–95).

>> We know of no research which has studied the contribution of eggs to whole-body composition in amphibians.

- There are some unclear/confusing aspects regarding juveniles/ unknown sex throughout the ms. Lines 184 – 185 – the sex does not need to change in the model, you should assign the correct sex to the whole history of the individuals once you know it, before analysis.

>> We did not assign sex “backwards” in encounter histories as small individuals were not only unsexed, but also were a separate age class, which were removed before analysis. Assigning a sex to small individuals would have no effect as they were not used in later analysis.

Lines 122-124 – “we removed juvenile Columbia spotted frogs from all analyses” state here the limits of the juvenile definition (SVL under which value). Are juveniles different from unknown sex? Then line 158, “We limited our inference on growth to adult frogs”, but state that in some cases unknown sex was changed to male or female, which suggests that some juveniles were included.

>> We have added language describing what SVL constitutes a juvenile Columbia spotted frog (131–132).

>> We marked and recaptured individuals as they grew across time, which sometimes included the transition from juvenile to adult. We limited our analysis to adult frogs, which included only portions of some animals’ encounter histories. This is the reason for the sex changing throughout an individual’s life (moving from “unknown sex” to male or female) and excluding juveniles, rather than entire individuals.

Minor comments
- Lines 5-7 – spell out numbers <10

>> Throughout the manuscript, we have changed to spelling out numerals <10, as is the PLOS standard.

- Lines 15- 18 – is this analysis done specifically for residual index? State here if so.

>> We have added that that these results are modeled for residual index (line 16).

- Rephrase line 29-30 “differences in fitness can scale up to population dynamics”

>> We have rephrased this sentence: “differences in fitness can affect population dynamics” (now line 31).

- Rephrase line 62-63

>> We have rephrased this sentence: “It is also unclear whether condition indices best represent percentage of body fat, or fat mass scaled to body size” (now 63–64).

- The fact that BCI has similar patterns in the 3 locations should be tested, either do some tests before analysis, or include the pond of origin as a factor in the models.

>> We did not have fully replicated sampling methodology (e.g., a full species x sites design) that would make a comparison of body condition across sites meaningful. Even if there was a difference in body condition index among the three sites, the relationship interest was the allocation of particular components (fat or lean mass) vs condition; light or heavy condition at a particular site would not necessarily change allocation patterns. An analysis of physiological differences in allocation based on environmental differences would be better ascertained by lab studies.

- Lines 81-87 – State here also that the growth study was done only in one of the locations, not only in the Fig. 1 legend.

>> We have added that language: “We used animals from all three sites to validate body condition indices, but animals from only one site (Jones Pond) to study growth” (83–84).

- Line 108 – include here that the animals were also released at capture site within 24 h.

>> We have added the fact that animals were held < 24h here (116).

- Lines 125-130 – is the scaling done separately for each sex? State here.

>> The scaling was across all sexes, as described by Peig and Green. Added language on line 136.

- Figs s1, s2, s4 – mention in legend what the prediction intervals represent. In s2, where no correlation exists, so remove the prediction interval

>> We have added information to each legend that the shaded intervals are 95% confidence intervals for the linear relationship. Keeping the confidence interval, *r*-value and *p*-value in S2, even where the relationship is near zero, is more illustrative for the reader.

- Lines 200-201 – are the differences significantly different?

>> Though we do not use formal significance tests for the difference in body fat by month, S4 includes 95% confidence intervals for the relationship between body fat and residual index, and the figure illustrates the general trend we mention in text.

- Table 2 – provide N for males also

>> We have added the N for males and females. The previous version mistakenly included the full sample size in females.

- Define at first mention, and then use BCI for body condition index consistently throughout the ms.

>> We intentionally chose to avoid this acronym because of its similarity to BMI, which caused confusion among early readers of the manuscript.

- Line 245-248 – this phrase should go in the discussion section, not in the results.

>> We feel that this phrase is better suited to the results section as it only reports results from the analysis. Interpretation of these results is included in the discussion paragraph beginning on line 330.

- Line 250 – replace “grew” with “had growth rate values between” or equivalent, to avoid the grew – 0.17 mm /day phrasing.

>> We have changed the language to match this suggestion (264–265).

- Line 251-252 - this phrase should go in the discussion section, not in the results

>> We felt it was important to include this point in the results, as it had little to do with our particular study and readers could be confused by the results if it were not mentioned immediately (265–267).

- Line 252-253 – delete “After removing body mass index due to high correlation with body length”, it is already in the methods. You can just put “(one for each BCI except BMI)” for clarity

>> We agree and have changed the language to match this suggestion (267).

- Rephrase line 284-285

>> We have rephrased the first sentence of the discussion for clarity (297–299).

- Line 371-373 – this comes out of the blue.

>> We have added language to tie this sentence to statements earlier in the paragraph: “by better understanding of an often-used indicator of fitness” (387-388).
