# Supplementary material for: The relationship between body condition, body composition, and growth in amphibians
Source: PLoS One. 2025 Apr 23;20(4):e0320954. doi: 10.1371/journal.pone.0320954 (PMC12061418; doi:10.1371/journal.pone.0320954)
Supplement: Attachment — Submitted filename: Response_to_Reviewers_auresp_2.docx [file pone.0320954.s004.docx]

Response to Reviewers

PONE-D-24-41754R1

The relationship between body condition, body composition, and growth in amphibians

PLOS ONE

Our responses to each reviewer point are set off by double-carats (>>) below. Line numbers refer to the “clean” manuscript version without tracked changes.

**Reviewer #1:** The manuscript is improved and most of the methodology is clear now. Nevertheless, I still have some comments.

1. regarding the questions on capture period for Rana luteiventris, in the section Response to reviewers you mentioned " while Columbia spotted frogs were captured June-August, well after they breed in our area" while in the response to reviewer 2 you state "while Columbia spotted frogs were captured during breeding and during June-August, well after they breed in our area." while in the M&M section, L92-93 we mention few during breeding and most afterwards.

>> Thank you for pointing out the inconsistency in our response to reviewers. In the response to reviewer #1, our response should have read “while Columbia spotted frogs were captured *during breeding and* during June-August, well after they breed in our area…”. The language in the manuscript on lines 91-93 is correct, the response to reviewer #2 is correct, and our justification in the response to reviewers still applies.

L16 and 18 - I suggest to refer only to faster growth rates and not provide the actual values.

>> We appreciate this suggestion, but providing estimated effect sizes in the abstract gives readers a better understanding of the biological significance of the findings. We also feel it is important to illustrate the difference in effect size between body condition and body length’s relationship to growth rate.

L19-21 should be moved at the beginning of the abstract

>> These lines are now at the beginning of the abstract (L 4-6).

L41-43 - Please rephrase.

>> We have restructured this sentence to make the point more clearly (now L 37–39).

L51 - quantities? Perhaps measures is better

>> Agreed, and changed the word “quantities” to “measures” (L 51).

L83 - Please rephrase, you are not validating the indices.

>> We have changed “validate” to “examine” in this sentence (L 83) and throughout the manuscript have used “examine” or “evaluate” instead (L 98, 203, 302)

L90 - visual encounter surveys is a method for inventory. Catching is done by hand.

>> We have changed “visual encounter surveys” to “hand capture” (L 90).

Figure 1 - I suggest moving it to supplementary online

>> Agreed, and this figure (study area map) has been moved to the supplementary material.

Table 1 - please check this paper that compared three body condition indices and make recommendations on what to use in amphibians
Băncilă, R. I., Hartel, T., Plăiaşu, R., Smets, J., & Cogălniceanu, D. (2010). Comparing three body condition indices in amphibians: a case study of yellow-bellied toad Bombina variegata. Amphibia-Reptilia, 31(4), 558-562.

>> Though this study had different goals than ours, we have added a citation in the discussion when discussing the relationship between condition indices and body size in amphibians (L 325).

L148-150 - you mention that the salamanders and treefrogs are abundant, but nevertheless the sample used is rather small. Why?

>> Long-toed salamanders and Sierran treefrogs are difficult to capture in this system. Both species are terrestrial for much of the year and their breeding season is somewhat unpredictable. Sierran treefrogs are highly cryptic and outside the breeding season, though males are more readily captured during breeding due to their calling behavior. We have added additional language to this point on L 147-148.

L211 - Which body condition? It is a bit unclear

>> We have moved this sentence to later in the results, and specified that we meant the relationship between body fat percentage and residual index varied by month in spotted frogs (L 237-239).

L299-300 - please state clear what BCI is better.

>> Though it would be helpful to mention a “best” BCI, our results show that sex, species, and body composition quantity of interest all change which BCI is best reflective of underlying composition. Rather than giving a simple recommendation in the first paragraph of the discussion, we feel the following paragraphs are important to explain the nuance in these relationships.

Discussion - I suggest to shorten the discussion and limit it to the usefulness of the study. Readers will be interested to see what BCI is recommended, what do they indicate and what are the limits of their use

>> As mentioned above, unfortunately there is not a clear recommendation for “best” BCI. Many studies in the past have been comparisons between indices with no discussion of the underlying quantities they reflect, or only focusing on a single species.
We feel that our study, and especially the discussion portion, is an important contribution to the literature surrounding body condition indices and their utility. We do make specific recommendations for BCIs that better reflect scaled fat quantities (L332-334).

L422, L477, L543 .... - italics for the scientific name

>> We have added italic Latin names to the works cited.

L511 - clamitans, italics

>> We have added italic Latin names to the works cited.

**Reviewer #3:** Overall, I agree with the previous reviewers that this study is valuable and will be a useful reference for future amphibian body condition research. In most cases, the authors have adequately addressed the previous reviewers’ comments. However, in a few cases further work is required, largely to provide clarity for readers. I have also included a small number of additional comments that should be addressed before publication.

Previous comments:

Both previous reviewers highlighted that there is an inadequate sample size for female Sierran treefrog (N = 4). Although this is acknowledged by the authors, conclusions and inference are still drawn from these differences. These inferences and conclusions are not supported based on four individuals and therefore, I suggest you only present overall results for this species in Table 4 and in the text (i.e. do not separate the numbers by sex).

>> We agree that the small sample size of female Sierrran treefrogs prohibits strong inference to that sex. However, evidence from all three species indicates that sexes may exhibit different patterns in the correlation of body components and body condition indices, which could make pooling sexes problematic. We have removed language from the conclusions where we report female treefrog results separately, and only include suggestions of possible sex-linked patterns, where appropriate, while also mentioning the small sample size (L 262–264, L 315–317). We have kept Table 4 separated by sex for consistency across all species, but added a note in the legend to further point out the small female sample size. We feel that, with sample size caveats, it is important to report these findings to potentially spur further research into the sex-linked nature of these relationships.

Reviewer 2 asked that BCI was defined at first mention, and then to use BCI for body condition index consistently throughout the ms. The authors state that: “we intentionally chose to avoid this acronym because of its similarity to BMI, which caused confusion among early readers of the manuscript.” However, the authors do use “BCI” extensively throughout the results and discussion section (e.g. L233, L235, L237, L238, L347). Whether acronyms are used or not are up to the authors, but consistency is needed in the ms.

>> Thank you for pointing out this inconsistency. We have updated our terminology throughout the manuscript, and only use “BCI” for clarity in tables and equations.

Line 211 and Figure S4: Reviewer 2 also commented on this result. Assessing whether body condition differed across months is not an aim of the paper, nor do the authors test this relationship statistically. Figure S4 does not provide a good visual representation of this result because it compares the residual index against percent fat when the comparison should be BCI against month. When focusing solely on the x axis (residual index) the mean residual index looks to be between -0.1 and 0.1 for each month, with some outliers for females and about 0.05 for males. It is not clear if the mean residual index differs between months. I suggest either removing this result and figure or incorporating this question into the manuscript fully. To incorporate it fully, ideally it would be done across the different BCIs (or at least SMI and residual index) and a formal test would be run with the BCI as the response metric and month as the predictor with ‘individual’ used as a random effect.

>> We agree that assessing differences in body composition across months was not an important aim of this paper. We only mention the difference in the relationship (slope in S4 figure) between percent fat and residual index as a general result (L 237–239; the *r*- and *p*-values in S4 figure illustrate this difference).

>> We have modified the sentence on L 377–379 to state that percentage body fat of female frogs was generally highest in August, though we did not statistically test this result: “female Columbia spotted frogs tended to have a higher percentage of body fat in August, compared to June or July (S4 Figure)”. We also provide 95% confidence intervals for the relationship in the S4 figure to better visually display differences.

Reviewer 2 commented on some unclear and confusing aspects regarding the use of juveniles/unknown sex throughout the ms. Regarding the “unsexed” individuals, the authors explain their reasoning in the response to reviewer document. However, this is still confusing in the ms. If juvenile measurements are excluded from all analyses, then Line 192-194 is not required (“we allowed sex to vary by time period because frogs may have grown enough between capture sessions to change from unknown to either adult male or female”) because the “unknown sex” measurements are excluded and only “male” or “female” are therefore contained in the dataset and analysis. Therefore, I suggest removing this sentence for improved clarity.

>> We have removed this sentence for clarity.

Additional comments:

Exclusion of juveniles: more justification is needed for removing juvenile individuals, especially for the body component section. The allometric relationship between length and mass is not expected to be linear which is the part of the basis behind the SMI and one of the arguments against the residual index. In the caption of Figure S2 it states: “Juvenile frogs were included in these correlations, but not in body composition analysis or growth models”. Does this mean that juveniles were included in the body condition index calculations but then excluded from on-going comparisons? Either way, this needs to be made clearer in the methods. Furthermore, the residual index and SMI use the relationship between the population mass and population length to calculate their BCI and if only a subset of the population was then used in the body component calculations this may affect the comparison between these measures. I suggest checking that this does not change the inference of your results, and to consider running a sensitivity analysis which does include juveniles in the body component and growth models.

>> We agree that allometry of the length-mass relationship across body sizes is not assumed for the SMI. However, we noted a difference in allocation of body fat as a percentage of mass between juvenile and adult Columbia spotted frogs (L 126-128; S2 Figure). This finding implies a life history difference in growth patterns that could potentially obfuscate important information about the relationship of body condition index to body components. Because we were not able to compare adults and juveniles across all three species, we removed juvenile Columbia spotted frogs from all analyses for consistency.

>> We did not include juvenile Columbia spotted frogs in the calculation of the residual index or scaled mass index. We added language to make this clearer on L 132.

>> We have removed the former S2 Figure from the supplemental material. We agree that it was confusing to portray the correlations of BCIs and SVL across all animals (including juveniles) when juveniles were not included in the analyses.

L179: 1 -> one. Also check captions, often using numeric symbols instead of words.

>> We have changed to spelling out numerals in this line (now L 176) and in figure captions.

L236: Here and elsewhere in the results the authors use the descriptors “weakly” “moderately” and “highly” but in some cases the R values overlap (e.g. L 246: Moderate = 0.48-0.65, High = 0.61 – 0.8. The authors should set the definition of these descriptors against values within the methods, I suggest near L157.

>> We have added a sentence defining our description of correlations (L 155–157) and now follow this convention in the results.

L251-253: This statement and a similar one in the abstract does not represent the results well and implies that BMI is the better BCI which is not a key result of this ms. Whether or not a BCI ranks better or worse than other BCI is not relevant in cases when all models perform badly. Perhaps more informative would be how often each BCI performed well (e.g. R> 0.6). This would be 5 times for BMI and 9 times for the other three.

>> We agree and have incorporated this suggestion into the abstract (L 13-16) and results (L 254-256).

L280-283: It is not clear why residual index and not SMI was used for these figures. This should be justified more explicitly, or the figures presented for both BCIs. I would prefer the later, because it aligns better with the overall theme of the ms. to compare BCIs.

>> We appreciate this suggestion. Results from the growth models of SMI and residual index were extremely similar (Table 5), and the plots were indistinguishable. We include a note in the legend for Figure 1 mentioning the similar results of the two models. We used residual index rather than SMI for the plots because of its broad use in the field, though we acknowledge that SMI would have been appropriate as well.

L337-339: The BCIs typically correlated well with scaled lean mass except for the large difference between male and female spotted frogs. It would be valuable to discuss potential reasons for this large difference between sex in this species.

>> We mention the potential for sex-linked differences in lean mass correlation with BCIs (L 337–342). We note that scaled lean mass was better reflected by BCIs in males of the two species for which we had sufficient sample size of both sexes. As for the marked difference between lean mass correlation in male and female Columbia spotted frogs, we do not think it appropriate in this study to further speculate on the reasons for that difference, besides the reasoning and reference given in lines 337–342.
